# Removal of Nitrogen, Phosphorus, Organic Matter, and Heavy Metals from Pig-Farming Wastewater Using a Microalgae-Bacteria Consortium

**DOI:** 10.1007/s00128-024-03962-2

**Published:** 2024-10-19

**Authors:** M. Sacristan de Alva, I. Oceguera-Vargas, E. Lamas-Cosío, K. León-Aguirre, F. Arcega-Cabrera

**Affiliations:** Unidad de Química en Sisal, Facultad de Química, Universidad Nacional Autónoma de México, Puerto de Abrigo S/N, Sisal, 97356 Yucatán México

**Keywords:** Microalgae, Microalgae-bacteria consortium, Wastewater, Pig farming, Metals

## Abstract

Wastewater generated by the pork industry urgently requires the implementation of low-cost, high-benefit, and efficient treatment systems. Accordingly, a microalgae-bacteria consortia-based treatment system is proposed for the removal of contaminants released, by the pork-producing industry, in swine wastewater. In this study, different inoculum concentrations of the microalgae-bacteria consortium were tested to document variation in the removal of nutrients from the wastewater. At varying concentrations, it was efficient and did not present a significant difference in the removal kinetics. The treatment with the greatest amount of inoculum removed close to 87% of total nitrogen, approximately 70% of orthophosphate, and 77% of chemical oxygen demand. Removals of 84% iron, 44% copper, and 48% manganese were also obtained. These results demonstrate that microalgae-bacteria consortia are an economically viable and environmentally desirable option for the efficient treatment of wastewater from the pork industry.

Industrialized pig farming generates wastewater that contains high loads of organic matter, ammoniacal nitrogen, and phosphorus, as well as heavy metals and chemicals from veterinary practices (Liu et al. 2022; Michelon et al. 2022). Thus, if these products are not treated adequately, they may generate serious environmental problems (Ferreira et al. 2021; Liu et al. 2022). Conventional treatment systems are neither economically viable nor environmentally sustainable. This is the case because they require large amounts of chemical compounds and energy, making them too costly. Furthermore, they contribute to climate change by releasing greenhouse gases into the environment (Chaudry 2021; Goswami et al. 2021; Morillas-España et al. 2021), while achieving only low removals of inorganic nutrients (e.g., phosphorus) (Fallahi et al. 2021).

Pork production, the second most consumed animal protein in the world, has increased in recent years. Lack of resources is one of the main causes of poor or no wastewater treatment (Chaudry 2021), since conventional wastewater treatment methods are usually expensive and inefficient (Nagarajan et al. 2019). Therefore, efficient, low-cost, and environmentally friendly treatment systems are needed; adoption of such systems would allow the industry to move towards greater sustainability (Liu et al. 2022). Currently, treatment systems are required that can purify wastewater, and at the same time capture nitrogen, phosphorus, and energy (in the form of organic carbon) contained in the wastewater for reuse in the manufacture of biofuels, biopolymers, biopesticides, and food supplements (Nagarajan et al. 2020; Goswami et al. 2021; Lee et al. 2021). The implementation of such strategies promotes a circular bioeconomy (Ferreira et al. 2021), within a concept of a biorefinery (Al-Jabri et al. 2021), where the use of biomass for commercial applications could represent extra income for pig farms (Ferreira et al. 2021).

Microalgae-based treatment offers one pathway to implementing needed systems. They have very simple nutritional requirements: carbon (organic/inorganic), nitrogen, phosphorus, and potassium that act as macronutrients; and trace metals that function as micronutrients (Markou et al. 2014). Taking advantage of photosynthesis (Li et al. 2023), they transform organic matter from wastewater into oxygen and biomass, also absorbing inorganic nutrients (Morillas-España et al. 2021), in addition to removing organic contaminants, heavy metals, and pathogens from wastewater (Suresh Kumar et al. 2015; Al-Jabri et al. 2021; López-Pacheco et al. 2021). The quantities of metals microalgae can absorb are influenced by various factors such as pH, the presence of co-cations (Shanab et al. 2012), temperature, the initial concentration of metals, the initial concentration of biomass, and time of contact (Qader and Shekha 2023). Microalgae have the ability to grow, assimilate, and resist toxic conditions, meaning that treatment systems based on microalgae can be integrated into existing systems or used as the only treatment method (Al-Jabri et al. 2021).

Microalgae and bacteria have mutual interactions and synergistic effects in the treatment system, so a microalgae-bacteria consortium helps to improve nutrient removal efficiency (Fallahi et al. 2021). Microalgae produce oxygen that is used by bacteria, which in turn produce secondary metabolites and CO_2_ used by microalgae (Herrera et al. 2021). Furthermore, microalgae can be beneficial for bacterial development, providing a physical space in which bacteria can protect themselves from adverse environments and release organic compounds that facilitate their growth (Gonçalves et al. 2017). Microalgae-bacteria consortia are beneficial because microorganisms aggregate, forming flocs that settle via gravity (Chia et al. 2023; Keshinro et al. 2024). So, biomass can be removed by means of a settling process. Treatment systems based on microalgae-bacteria can provide energy savings of around 85% of the total costs associated with an activated sludge system (de Godos et al. 2017). These characteristics provide profitable returns to treatment systems based on microalgae-bacteria consortia in terms of nutrient recovery due to their lower energy requirements and less environmental impacts (Ferreira et al. 2021).

The present work focused on growing a consortium of microalgae-bacteria in wastewater from a pig farm by adding different quantities of inoculum of microalgae to the wastewater. Then, the efficiency of the microalgae and bacteria consortium was evaluated in terms of nitrogen, phosphorus, organic matter and heavy metals removal from wastewater. The goal was to develop an assessment of a wastewater treatment system that can efficiently remove pollutants. Thus, the treated water can be reused, depending of the quality obtained (but at least for washing pens), reducing water extraction. At the same time, products can be obtained from the biomass making the system economically viable. Accordingly, pig farms could viably implement it, reducing the pollution of water bodies. Thereby, the study evaluated its efficiency, economic viability, and environmental relevance.

## Materials and Methods

Microalgae-bacteria consortia was isolated using an Instituto Tecnológico de Conkal (Yucatán, México) pig farm’s oxidation lagoon. The consortium was acclimated to wastewater by growing it in a medium with 75% by volume of Peters 20-20-20 ^®^ fertilizer and 25% wastewater. Every week,1/3 of the crop was removed, and wastewater was added. This was carried out for 6 weeks (Osundeko et al. 2014). The wastewater (dark brown in color and odorless), from a primary treatment (consisting of a settling process) and a secondary treatment based on anaerobic treatment, was obtained from an intensive pig farm (producing near 45,000 pigs per production cycle) in the state of Yucatán, Mexico.

The wastewater was characterized by measuring nitrate (NO_3_^−^), nitrite (NO_2_^−^) (Miranda et al. 2001), phosphate (PO_4_^3−^), ammonium (NH_4_^+^) (Hernández-López and Vargas-Albores 2003), and chemical oxygen demand (COD) (Bridgewater et al. 2017). Total nitrogen (TN) was obtained from the sum of NO_3_^−^, NO_2_^−^ and NH_4_^+^. Several important quality parameters like dissolved oxygen (DO), pH, temperature (T), conductivity (EC), salinity (S), and total dissolved solids (TDS) were measured using a YSI ProQuatro probe, previously calibrated based on the manufacturer’s guidelines.

The consortia were cultured in 2 L Erlenmeyer flasks containing 500 mL of wastewater and 1000 mL of inoculum. The culture conditions included a photoperiod of 12 h:12 h (light: dark) with a photosynthetically active photon radiation of 750 µmol/m^2^s provided by a LED light, constant aeration to maintain the culture in agitation, and a temperature of 25 ± 2 °C.

Four treatments were carried out with different concentrations of inoculum (obtained from the consortium grown in wastewater): Treatment A contained 500 mL of wastewater and 1300 mL of inoculum; B had 500 mL of wastewater and 1200 mL of inoculum; C involved 500 mL of residual water and 1100 mL of inoculum; and D had 500 mL of residual water and 1000 mL of inoculum. The inoculum was obtained from microalgae growing in the same wastewater used in this research. These cultures were performed in triplicate. There was a control of uninoculated residual water under the same conditions (Control).

The wastewater was characterized at days 0, 2, 4, 7, 9, 11, 14, and 16 (the data are presented in the supplementary material) by taking 10 mL of sample and filtering it through 0.45 μm MF-Millipore MCE membranes. Determination of metals was conducted by taking an aliquot of 9 mL from each experiment. Every sample was subjected to microwave oven-assisted acid digestion, using an Anton Paar Synthos 3000, with HNO_3_ (Suprapur Sigma Aldrich 70% redistilled > 99.999% metals basis). Metals were measured by atomic absorption spectroscopy, using a Perkin Elmer Aanalyst 800 atomic absorption spectroscoper, in water samples (following the filtration process mentioned above) taken at day 0 and at day 11 of the culture (Arcega-Cabrera and Fargher 2016; Arcega-Cabrera et al. 2021). The removal kinetics were determined according to Wang et al. (2014) and the removal percentages according to Nguyen et al. (2022). Statistical differences among treatment data sets were determined using one-way analysis of variance (ANOVA) with OriginPro 2019b v9.6.5.169 (OriginLab Corporation) Tukey’s significant difference test was conducted after the determination of variances (*p* ≤ 0.05).

## Results and Discussion

The initial concentrations of contaminants in the wastewater from the pig farm are reported in Table 1. Due to the dark brown color (low light penetration) of the wastewater, a larger volume of microalgae inoculum was used to favor its growth. The lower initial concentration of the contaminants in experiments A, B, C, and D with respect to the control was due to the addition of the microalgae inoculum. At the beginning of the culture, the residual water had a pH of 8.32 and 5616 mg/L of dissolved solids. A reduction was observed in both the pH values and total dissolved solids in both the treatments and the control (Table 1). The reduction in pH may have been due to the presence of bacteria in the wastewater or the use of ammonium by microalgae that, to maintain cellular neutrality, release protons by absorbing ammonium (Eustance et al. 2013).

**Table 1:** Removal percentages and removal kinetics of ammonia, nitrate, nitrite, orthophosphate, and COD

	A	B	C	D	Control
NH_4_^+^ (mg/L)					
Initial	381.62 ± 2.51	395.35 ± 7.12	408.34 ± 3.49	437.83 ± 2.07	757.66 ± 4.59
Day 11	62.56 ± 5.85	76.16 ± 7.02	108.15 ± 4.00	108.95 ± 4.13	3.17 ± 1.01
Removal percentage	**83.61**	80.75	73.51	75.12	99.58
Removal kinetics	29.01	29.02	27.29	29.90	68.59
NO_3_^−^ (mg/L)					
Initial	556.38 ± 9.42	573.84 ± 4.50	592.66 ± 3.13	615.54 ± 3.11	935.27 ± 3.73
Day 11	81.44 ± 8.92	94.50 ± 7. 26	108.53 ± 7.22	114.69 ± 3.39	1817.15 ± 4.39
Removal percentage	**85.38**	83.54	81.68	81.37	− 94.29
Removal kinetics	43.18	43.58	44.01	45.53	− 80.17
NO_2_^−^ (mg/L)					
Initial	183.44 ± 4.93	190.62 ± 6.71	203.62 ± 5.06	218.27 ± 4.26	462.16 ± 3.60
Day 11	1.85 ± 0.55	6.82 ± 0.21	34.96 ± 4.87	20.50 ± 4.51	338.16 ± 10.16
Removal percentage	**99.00**	96.42	82.80	90.63	26.84
Removal kinetics	16.51	16.71	15.33	17.98	11.27
Total nitrogen (mg/L)					
Initial	1121.45 ± 6.47	1159.81 ± 14.92	1204.61 ± 6.70	1271.63 ± 6.45	2155.09 ± 10.38
Day 11	145.84 ± 14.23	177.48 ± 13.40	251.64 ± 12.06	244.14 ± 3.95	2158.48 ± 14.57
Removal percentage	**87.00**	84.70	79.11	80.80	− 0.16
Removal kinetics	88.69	89.30	86.63	93.41	− 0.31
PO_4_^3−^ (mg/L)					
Initial	6.06 ± 0.07	6.71 ± 0.22	7.77 ± 0.18	8.53 ± 0.09	9.77 ± 0.08
Day 11	1.84 ± 0.12	3.08 ± 0.10	2.92 ± 0.09	3.44 ± 0.07	9.47 ± 0.02
Removal percentage	**69.68**	54.13	62.36	59.69	3.07
Removal kinetics	0.38	0.33	0.44	0.46	0.03
COD (mg/L)					
Initial	224.60 ± 4.54	261.48 ± 6.40	312.08 ± 6.71	360.71 ± 7.11	536.20 ± 13.26
Day 11	51.40 ± 1.07	52.45 ± 0.59	66.39 ± 3.59	60.20 ± 2.94	346.50 ± 12.57
Removal percentage	77.11	79.93	78.73	**83.30**	35.39
Removal kinetics	15.75	19.00	22.34	27.32	17.25
pH					
Initial	8.06 ± 0.07	7.75 ± 0.09	8.13 ± 0.11	8.18 ± 0.21	8.32 ± 0.22
Day 11	5.83 ± 0.13	5.61 ± 0.17	6.20 ± 0.12	6.33 ± 0.19	6.12 ± 0.24
Total dissolved solids (mg/L)					
Initial	4836 ± 251	4745 ± 211	4595 ± 132	4736 ± 151	5616 ± 97
Day 11	3246 ± 115	3308 ± 173	3165 ± 112	3433 ± 123	3841 ± 113
Removal percentage	32.88	30.28	31.12	27.51	31.61

Highest removal percentage in bold font. The values are means (*n* = 3) ± standard deviation

**Fig. 1:**
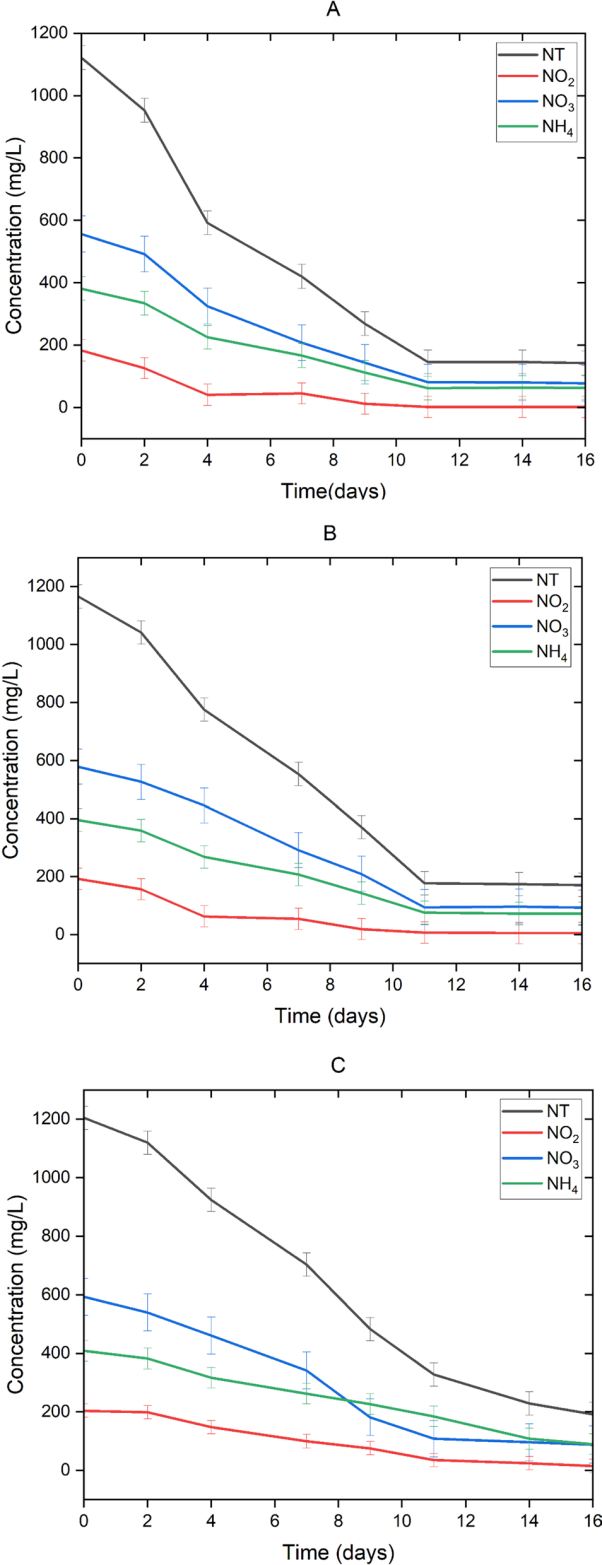

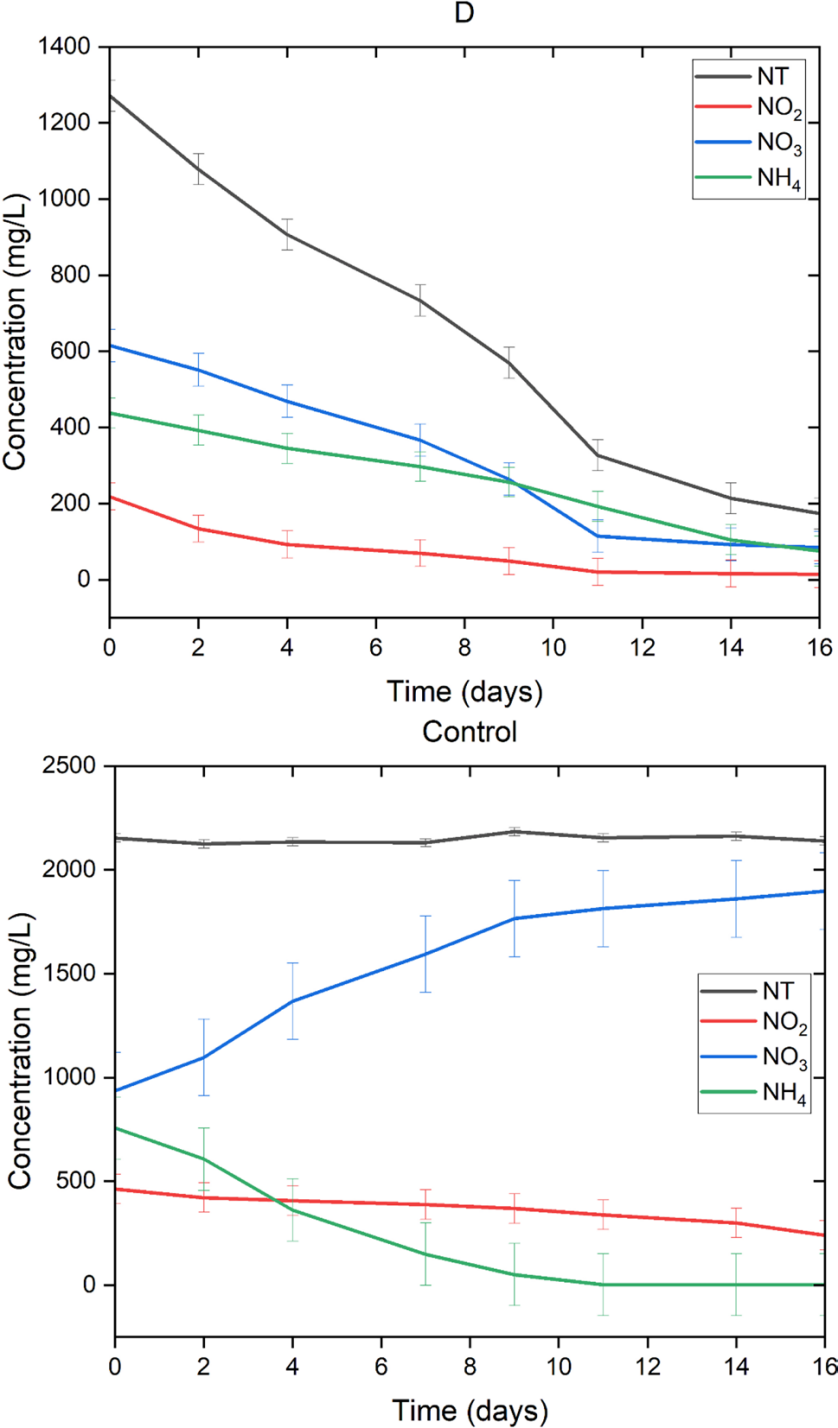
Nitrogen (ammonia, nitrate, nitrite, and total nitrogen (TN)) concentration in the four different cultures and the control. The graphed data are the means of replicates (*n* = 3) ± standard deviation bars with a scaling factor of 0.5

The highest percentage and removal kinetics of ammonium are achieved in the Control. However, this is probably due to it being oxidized and transformed into nitrate due to the presence of microorganisms in aerobic conditions (Lawson and Lücker 2018). So, the reduction of ammonium is not because of the removal, but because of its transformation, since the concentration of total nitrogen remains almost constant (Fig. 1). Something similar happens with nitrite, although in a smaller proportion (Fig. 1). This is the reason why the percentage and kinetics of nitrate removal are negative in the Control (Table 1).

The greatest removal of NH_4_^+^, NO_2_^−^, NO_3_^−^ and PO_4_^3−^ occurred in Treatment A. In the case of COD and TDS the greatest removals were achieved in Treatment D (Table 1). No significant difference among treatments (*p* > 0.05) was found for NO_2_^−^, NO_3_^−^ and PO_4_^3−^ removal. Only ammonium and COD removals presented significative difference (*p* < 0.05). This could be because ammonium is the form of nitrogen preferred by microalgae, so if the inoculum of microalgae is larger, ammonium will be consumed faster. Also, if the inoculum is greater, is expected that more oxygen is being produced by the microalgae, so aerobic bacteria could oxidize ammonium to nitrate, increasing its removal kinetics. Finally, bacteria remove organic matter efficiently due to the oxygen supplied by microalgae (Aditya et al. 2022).

In all cases, the percentage of nutrient removal by the microalgae-bacteria consortium becomes constant after day 11 (Fig. 1A, B, C, and D), therefore nutrient removal and removal kinetics were calculated between day 0 and day 11 (Table 1). Moreover, the removal percentage and kinetics were considered until day 11 because treatments at this lapse of time are more economical than ones of 14 or 16 days. To determine the difference in removal percentages between day 11, 14 and 16, a Tukey test was performed. In the case of Treatment A, Tukey test showed no significant difference (*p* > 0.05) among the removal percentages on day 11, 14, and 16. In the case of B, there is only a significant difference (*p* < 0.05) in the nitrite removal percentages. In the case of C, there is only a significant difference (*p* < 0.05) in the percentages of total nitrogen removal. In the case of D, there is a significant difference (*p* < 0.05) in the percentages of ammonium, nitrite, and total nitrogen removal. Orthophosphate and COD removal do not show any significant differences (*p* > 0.05) for the removal percentages in any of the cases.

The removal of total nitrogen by the consortium for day 11 in treatment A is 79.1%, which is lower than that reported for the treatment of swine wastewater by *Chlorella vulgaris* (90%), *Chlorella zofingiensis* (85%), and *Scenedesmus obliquus* (95%) (Cheng et al. 2019). In these studies, pure strains are used for the treatment of wastewater with a prior treatment that excludes the bacteria present, limiting the reproducibility of the operating conditions by avoiding the formation of consortia of microorganisms (Foladori et al. 2018). In the case of *C. vulgaris*, filtration occurs in the residual water; whereas, in the case of *C. zofingiensis* and *S. obliquus*, the water is autoclaved and diluted. These prior treatments would increase water treatment costs, in addition to making the treatment process less environmentally friendly. The reason for the high removal efficiencies as compared with this study is due to the initial nitrogen concentrations being low, e.g., 56, 289, and 148 mg/L for *C. vulgaris*, *C. zofingiensis*, and *S. obliquus* respectively, this is because at similar times lower concentration of nutrients could be achieved when the initial concentration is also lower.

In all cases, greater nutrient removal kinetics were observed in Treatment D than in A. This is because in Treatment D there is a greater amount of nutrients due to the addition of a smaller amount of inoculum, so the dilution because of the inoculum addition is greater in treatment A than in treatment D. This also explains why the lowest nutrient concentrations in the wastewater at day 11 of culture were observed in Treatment A (Table 1). The difference in removal percentages in the treatments may have been due to the initial concentration of nutrients in the culture and to the competition among microalgae and other microorganisms present in the wastewater (Condori et al. 2023).

**Fig. 2:**
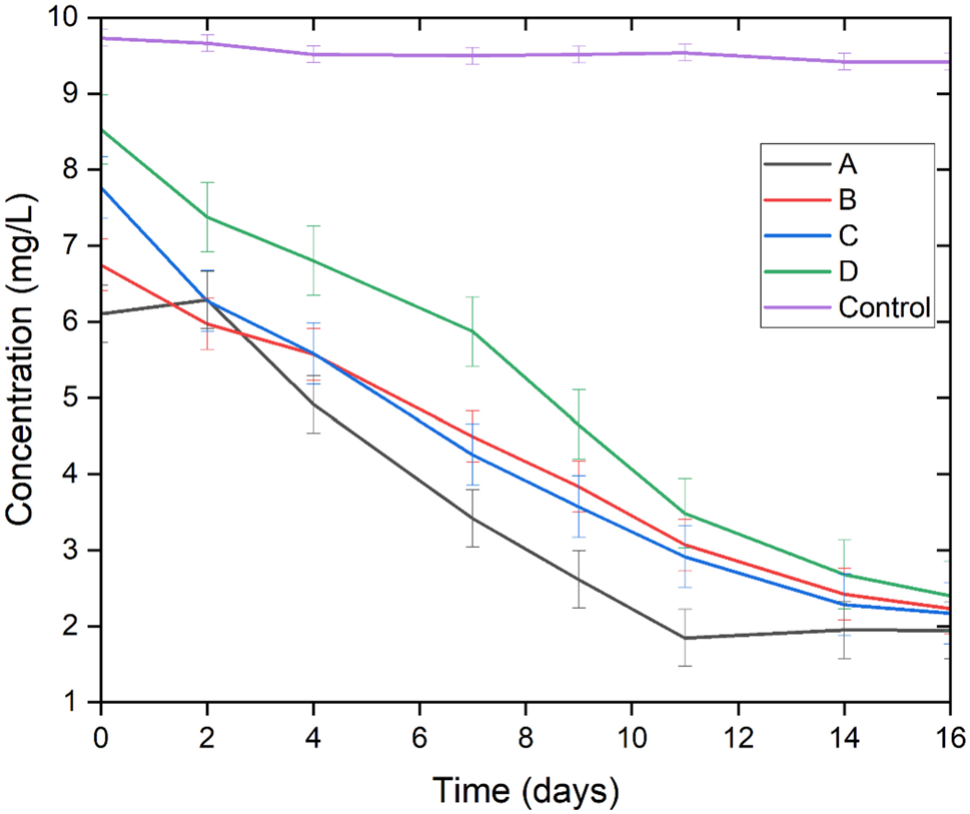
Phosphate concentration in the four different cultures and the control. The graphed data are the mean of replicates (*n* = 3) ± standard deviation bars with a scaling factor of 0.5

In Fig. 1, a similar behavior can be observed in the four treatments. Among the treatments, there is no significant difference (*p* > 0.05) for the removal percentages or for the kinetics of nitrate removal. This is, probably, because nitrate is the form of nitrogen that is found in the greatest proportion. Because ammonium is consumed and/or oxidized in the first days, nitrate is the form of nitrogen that consortium will absorb in the consecutive days (Taziki et al. 2015) (Fig. 2).

The significantly different (*p* < 0.05) removal kinetics of ammonium among treatments, may be related to the different initial responses on microalgae to ammonium. Because of this, it is expected that there will be a period of delay in the removal of ammonium (Chai et al. 2021).

Table 2 shows the initial concentration of metals (day 0) present in the wastewater, as well as the removal percentage obtained, after 11 days of treatment.

**Table 2:** Concentrations of metals in the water for treatments and control

	A	Mean removal (%)	B	Mean removal (%)	C	Mean removal (%)	D	Mean removal (%)	Control	Mean removal (%)
Mg (mg/L)	16.78 ± 0.29	0.0	15.64 ± 0.81	0.0	14.89 ± 0.38	0.0	13.98 ± 1.06	0.0	14.88 ± 0.86	0.0
Pb (µg/L)	ND	0.0	ND	0.0	ND	0.0	ND	0.0	ND	0.0
Ni (µg/L)	11.72 ± 0.54	1.68	11.14 ± 0.92	1.46	11.42 ± 1.71	4.50	9.67 ± 1.86	9.58	15.02 ± 1.06	12.14
Cd (µg/L)	0.10 ± 0.04	0.0	0.10 ± 0.03	0.0	0.09 ± 0.01	0.0	0.08 ± 0.02	0.0	0.17 ± 0.02	0.0
Zn (mg/L)	0.20 ± 0.09	14.17	0.21 ± 0.07	6.03	0.18 ± 0.01	6.53	0.17 ± 0.04	6.60	0.09 ± 0.02	7.48
Cu (mg/L)	0.11 ± 0.02	44.17	0.10 ± 0.01	40.14	0.10 ± 0.01	39.97	0.09 ± 0.02	43.19	0.10 ± 0.02	17.08
Fe (mg/L)	0.24 ± 0.03	84.81	0.22 ± 0.02	92.05	0.22 ± 0.01	99.99	0.20 ± 0.01	99.99	0.12 ± 0.04	99.99
Mn (mg/L)	0.213 ± 0.014	48.120	0.201 ± 0.011	34.410	0.180 ± 0.002	54.434	0.178 ± 0.026	54.472	0.125 ± 0.019	0.0

*ND* non detected

The only metal that was removed in 99.99% of the water was iron, probably because it is an essential micronutrient for microalgae (Rana and Prajapati 2021). However, it was also removed in the control, probably by iron-oxidizing bacteria that could be present in wastewater (Aziz et al. 2020). Manganese and copper were removed to a lesser extent. Manganese had an approximate removal of 50% except in Treatment B; it is also an essential micronutrient for microalgae (Liu et al. 2022), in this case there was no removal in the control. Copper, which is toxic to microalgae at high concentrations, had an approximate removal of 40%; in the control, the removal was only 17%. A low removal of zinc was also obtained, only about 14% in Treatment A and about 6% in the other treatments. These values are similar to 7% removal in the control, so it seems that microalgae has nothing to do with its removal. Regarding nickel, there is greater removal in the control than in the treatments. Cadmium is present in low concentrations in wastewater and its removal was not observed. The other metal that was not removed is magnesium, of which some removal was expected since it is necessary for chlorophyll. The low removal of some metals may be due to the fact that they are associated with solids in the water. Thus, they are not dissolved (Huang and Wang 2001) and are not bioavailable. Furthermore, the removal is affected by several factors, such as the initial concentration of metals (Manzoor et al. 2020), pH, temperature (Manzoor et al. 2020; Ahmed et al. 2022), the source of water (Abdel -Razek et al. 2019), and biomass productivity (Ahmed et al. 2022). Due to these reasons metal removal is lower than conventional methods that, in general, can achieve removals greater than 80%, nevertheless conventional processes are efficient only at high initial metal concentrations and are very costly compared with microalgae removal (Razzak et al. 2022).

Lower copper removals were obtained than those obtained by *C. pyrenoidosa* in municipal wastewater, where removal reached 60% (Kothari et al. 2022). In the case of manganese, removals were around 75%, higher than those reported in this study. Zinc and nickel removals achieved by *C. pyrenoidosa* (Kothari et al. 2022) were close to 60%, much higher than those obtained in the present study.

The energy consumption of the reactors was calculated according to Singh et al. (2012). The total energy consumed to carry out the wastewater treatment was 0.23 kWh/m^3^, considering the consumption of the stirring equipment, lighting, and temperature control. This consumption is lower than that of conventional treatment systems such as activated sludge, which require between 0.5 (Acién et al. 2016; de Godos et al. 2017) and 0.6 kWh/m^3^ (Morais et al. 2021). On average, 0.92 ± 0.07 g/L of dry biomass was obtained, therefore the energy consumed per kg of biomass is 0.25 kWh/kg, considering harvesting and drying processes. This is less than the energy required by Gouveia et al. (2016), which was between 0.33 and 0.36 kWh/m^3^ and 0.32 kWh/kg respectively. Thus, scaling the reactors and keeping them in ambient conditions could in turn reduce energy consumption created by the artificial light source and temperature maintenance in these experiments.

Results demonstrate that microalgae and bacteria consortia are an effective option for reducing contaminants because they tolerate wastewater conditions without pretreatment, as well as effectively remove nitrogen (> 80% reduction), orthophosphate (> 50% removal), and some metals (e.g., Cu, Fe, and Mn). The inoculum concentration affected the nutrient removal percentages % and greater than 80%, respectively. The inoculum concentration affected the nutrient removal percentages, having the greatest removal percentages in the treatment with the greatest inoculum concentration. However, it did not have a significant effect on removal kinetics. In the case of the treatment with the greatest inoculum there was no significative difference in the removal percentage at the end of the exponential phase than in the stationary phase. This indicates that there is no need to extend cultivation time, which would increase treatment costs. This research constitutes a significant advance in developing effective, low-cost wastewater treatment for the pig industry and could be replicated elsewhere with a consequent positive environmental impact. Furthermore, the treatment with a microalgae-bacteria consortia demonstrated efficacies that are comparable with or exceed, in some cases, those of traditional water treatment processes but at much lower cost. Thus, the results indicate that the consortia evaluated in this research could contribute to making wastewater treatment more economically viable for the pig-farming industry, which, if broadly adopted, would result in positive socio-environmental impacts. More research is needed to achieve greater metal removal and to lower treatment time to reduce costs. These could be achieved by a semicontinuous or continuous culture of the consortium.
